# An Effective Hybrid Self-Adapting Differential Evolution Algorithm for the Joint Replenishment and Location-Inventory Problem in a Three-Level Supply Chain

**DOI:** 10.1155/2013/270249

**Published:** 2013-12-17

**Authors:** Lin Wang, Hui Qu, Tao Chen, Fang-Ping Yan

**Affiliations:** ^1^School of Management, Huazhong University of Science and Technology, Wuhan 430074, China; ^2^College of Public Administration, Huazhong University of Science and Technology, Wuhan 430074, China

## Abstract

The integration with different decisions in the supply chain is a trend, since it can avoid the suboptimal decisions. In this paper, we provide an effective intelligent algorithm for a modified joint replenishment and location-inventory problem (JR-LIP). The problem of the JR-LIP is to determine the reasonable number and location of distribution centers (DCs), the assignment policy of customers, and the replenishment policy of DCs such that the overall cost is minimized. However, due to the JR-LIP's difficult mathematical properties, simple and effective solutions for this NP-hard problem have eluded researchers. To find an effective approach for the JR-LIP, a hybrid self-adapting differential evolution algorithm (HSDE) is designed. To verify the effectiveness of the HSDE, two intelligent algorithms that have been proven to be effective algorithms for the similar problems named genetic algorithm (GA) and hybrid DE (HDE) are chosen to compare with it. Comparative results of benchmark functions and randomly generated JR-LIPs show that HSDE outperforms GA and HDE. Moreover, a sensitive analysis of cost parameters reveals the useful managerial insight. All comparative results show that HSDE is more stable and robust in handling this complex problem especially for the large-scale problem.

## 1. Introduction

As a multiitem inventory replenishment policy, the joint replenishment (JR) which can save the total costs by grouping multiitems in the same order had received numerous attentions [1]. While research on the classic joint replenishment problem (JRP) reached a saturation point, many extension versions had been proposed. These extensions can be divided into two branches: (a) relax the assumptions (such as storage, demand, and capacity limitation) of the classic JRP to simulate more realistic problems. The extensive literature review is available in [1]; (b) integrate more supply chain decisions (strategic or operational).

Silva and Gao [2] pointed out that three important decisions, facility location decisions, inventory management decisions, and distribution decisions, are highly related within a supply chain. Although independent policy always leads to a degree of suboptimization [3], these decisions usually are undertaken separately due to the complexity of integration. The existing works of literature were mainly the any two of three decisions integration. Wang et al. [4] proposed a two-level joint replenishment and delivery problem (JRD) by extending the model of Qu et al. [5]. The comparative results verified that the hybrid differential evolution (HDE) algorithm is effective. Cha et al. [6] considered the JRD of a three-level supply chain. A heuristic algorithm and an intelligent algorithm were proposed to solve this JRD. An extension of this JRD by adopting the freight consolidation policy was proposed by Moon et al. [7]. All the papers mentioned above were the integration of inventory and distribution decisions within one warehouse or distribution center (DC). The cost-saving can be achieved by sharing fixed ordering cost and transportation cost and economies of scale. With the development of global purchasing and supply chain management, there had been a strong move towards integration of location and inventory decisions between multi-DC (Berman et al. [8]).

Except Berman et al. [8] and Silva and Gao [2], the early studies on location-inventory problems (LIPs) had almost exclusively assumed the continuous-review (*r*, *Q*) inventory policy. Teo et al. [9] formulated a LIP ignoring the shipping costs from the DCs to retailers. Daskin et al. [10] studied a LIP including location cost, inventory related costs, transportation costs from the supplier to the DCs, and local delivery costs from the DCs to the retailers. They proposed a Lagrangian relaxation solution algorithm for the proposed model. Shen et al. [11] considered the similar problem of Daskin et al. [10] by restructuring the model as a set-covering model and used column generation to solve the model. Based on the work of Daskin et al. [10] and Shen et al. [11], several works of literature had been published on this topic [3, 12–14].

The periodic-review (*R*, *S*) policy was introduced into the LIP by Berman et al. [8]. They pointed out that the (*R*, *S*) policy is easier to coordinate than the (*r*, *Q*) policy and integrating the JR policy is a possible direction for future research. Silva and Gao [2] firstly proposed a LIP by considering the JR policy simultaneously. They solved this model using a two-stage method, the first stage, a Greedy Randomize Adaptive Search Procedure (GRASP) was used to determine the location decision; the second stage solved the JRP corresponding to the locations defined in the first stage. The proposed method is more suitable for the cases of specified number of opened DCs. This is the only one paper about the joint replenishment and location-inventory problem (JR-LIP). Thus it is meaningful to extend the JR-LIP and provide a new effective solving approach.

JRPs and uncapacitated facility location problems had been proven to be the NP-hard problems and they were rather hard to find effective algorithms [15, 16]. Current solution approaches for LIPs included different heuristics based on the complex mathematical analysis of the models, that is, Lagrangian relaxation based solution [10, 12], column generation algorithm [3, 11], conic integer programming approach [17, 18], and greedy randomized adaptive search procedure [2]. However the problem becomes much more complex than traditional LIPs because of the introduction of the JR policy (the ordering frequency of each DC and the basic ordering time needed to determine). It is rather hard to solve this JR-LIP effectively by traditional approaches. Firstly, available heuristics are too problem-specific and rather difficult to design. There exists no versatility approaches. Secondly, the enumeration is inefficient. It may take years to find a solution for a large problem size. On the other hand, due to the advantage of ignoring the mathematical property to search the optimal solution by starting from a feasible solution, intelligent algorithms had grown quickly in handling JRPs and JRDs. Results of similar studies illustrated that genetic algorithms (GAs) and differential evolution algorithms (DEs) are comparable suitable approaches [4, 19–22]. Although the existing works of literature hinted the superior of ant colony optimization (ACO) in solving combinatorial optimization, Wang et al. [22] pointed out that ACO was inferior in convergence rate for the similar JRD and more difficult to be expanded to complex problems.

The aim of this study is to propose a new and effective approach to handle the modified JR-LIP model based on the study of Silva and Gao [2]. The differences between our model and Silva and Gao [2] are as follows: (I) we assume that the maximum number of DC is known (additional variables were added in our model), the objective is to determine the number of DC to be opened, the locations of these DC, the assignment of customers, the replenishment frequency of each DC, and the replenishment cycle time to minimize the total system cost; (II) in Silva and Gao [2], the optimal number of DCs is obtained through contrasting the total cost by ADDING (or DROPPING) DC one by one. This method is not suitable for large-scale problems. The proposed hybrid self-adapting DE (HSDE) can directly find the optimal number of DCs through an intelligent search in a given space and is relatively easier to extend for large-scale problems. In fact, the HSDE can overcome the disadvantage of one-to-one competition used by classic DE and can avoid the manual parameter testing of mutation factor and crossover factor. Results of benchmark functions tests and numerical examples show the robustness of the HSDE.

The rest of this paper is organized as follows.  Section 2 proposes the mathematical model. In Section 3, a DE-based algorithm is proposed to solve the JR-LIP.  Section 4 is numerical examples and results discussion.  Section 5 contains the conclusions and future research directions.

## 2. The JR-LIP Model and Analysis

### 2.1. Assumptions and Notations

A three-level supply chain consisting of multidistribution centers (DCs), an outside supplier, and multicustomers is considered. The item is ordered and collected by the DCs and then is distributed to customers. The objective is to decide the following policy: (1) how many DCs should be opened, and where to locate them; (2) how the customers are assigned to appropriate DCs; (3) how many and when to order, to minimize the total cost. The JR-LIP model is studied based on the following assumptions.

- Demand rates and costs are known and constant.
- Shortages are not allowed.
- Replenishment lead time is constant.
- Each customer is only assigned to one DC, while other DCs cannot serve it.
- There is no limitation for the capacity of storage and shipment.

**Figure 1:**
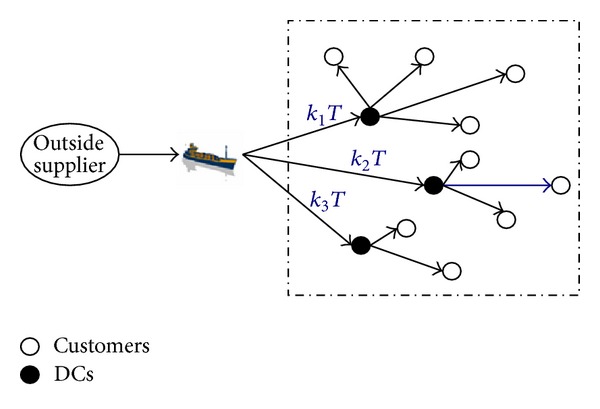
The three-level supply chain of proposed JR-LIP.

Only one item is considered in our model, DCs replenish their demand jointly. DC *i* replenishes its item at every integer multiple *k*_*i*_ of the basic cycle time *T* and then delivers them to customer *j*.  Figure 1 gives a simple description about our model.

Notions used in the model are as follows:

 
*i*: index of DCs, 1 ≤ *i* ≤ *n*;
 
*j*: index of customers, 1 ≤ *j* ≤ *m*;
 
*l*: index of potential sites for DC, 1 ≤ *l* ≤ *m*;
 
*D*_*j*_: annual demand rate for the item per unit time at customer *j*;
 
*S*: DCs' major ordering cost;
 
*s*_*l*_: minor ordering cost of potential DC site *l*;
 
*h*_*l*_: annual inventory holding cost of item at potential DC site *l*;
 
*c*_*lj*_: the Euclidean distance between potential site *l* and customer *j*;
 
*f*_*l*_: the fixed cost of opening a DC at potential site *l*;
 
*T*: basic cycle time (decision variable);
 
*k*_*i*_: ordering cycle time of item at DC *i*, integer number (decision variable);
 
*Z*_*i*_: the variable to decide whether DC *i* to be opened (decision variable), it is defined as
  (1) $$\begin{array}{c} Z_{i}=\{\begin{array}{cc} 1, & \text{if}\;\;\text{DC}\;i\;\;\text{is}\;\;\text{opened},\\ 0, & \text{otherwise}; \end{array} \end{array}$$
 
*Y*_*il*_: the location variable (decision variable), it is defined as
  (2) $$\begin{array}{c} Y_{il}=\{\begin{array}{cc} 1, & \text{if}\;\;\text{DC}\;i\;\text{is}\;\;\text{located}\;\;\text{at}\;\;\text{potential}\;\;\text{site}\;\;l,\\ 0, & \text{otherwise}; \end{array} \end{array}$$
 
*X*_*ij*_: the assignment variable (decision variable), it is defined as
  (3) $$\begin{array}{c} X_{ij}=\{\begin{array}{cc} 1, & \text{if}\;\text{customer}\;j\;\text{is}\;\;\text{assigned}\;\;\text{to}\;\;\text{DC}\;i,\\ 0, & \text{otherwise}. \end{array} \end{array}$$

### 2.2. Mathematical Model and Analysis

The total cost is composed of the fixed location costs of DCs, assignment costs of DCs, and replenishment costs of DCs. For a given problem, we assume that the maximum number (*n*) of DCs is known and each site of customer is a potential site for a DC.

*(a) Location Costs*. The distance between customers is not considered in our model. Thus, besides the effect of replenishment policy, the main concern for making the location decision is the fixed opening cost and the distances from DCs to customers which are usually used to measure transportation costs in a supply chain network [2, 4]. So the total location costs (*C*_*L*_) can be written as

(4) $$\begin{array}{c} C_{L}=\overset{n}{\underset{i=1}{\sum }}\;\overset{m}{\underset{l=1}{\sum }}f_{l}Y_{il}Z_{i}+\overset{n}{\underset{i=1}{\sum }}\;\overset{m}{\underset{l=1}{\sum }}\;\overset{m}{\underset{j=1}{\sum }}c_{lj}X_{ij}Y_{il}Z_{i}. \end{array}$$

In (4), the first term is the fixed cost of locating DCs. The second term is the transportation cost of shipping from DCs to customers.

*(b) Replenishment Costs*. Similar to the classic JRP, the replenishment costs of DCs include ordering costs which consist of major ordering cost and minor ordering cost and inventory holding cost. The difference is the frequency of orders and the basic ordering cycle time at each DC is determined by the demand served by the DC. In turn, the demand served by the DC is a function of the assignment of customers to the DC. Thus the annual replenishment costs (*C*_*R*_) are formulated as

(5) CR=ST+∑i=1n ∑l=1mslkiTYilZi+∑i=1n ∑l=1mhlkiTDi2YilZi, 

where *D*_*i*_ = ∑_*j*=1_^*m*^ *D*_*j*_ *X*_*ij*_ is the demand for the item by unit of time allocated to DC *i*. The first term is the major ordering cost of DCs; the second term is the total minor ordering cost of DCs; the third term is the annual inventory holding cost of DCs in (5).

*(c) The Objective*. According to the above analysis, the annual total cost (TC) is

(6) TC=CL+CR=∑i=1n ∑l=1mflYilZi+∑i=1n ∑l=1m ∑j=1mcljXijYilZi +ST+∑i=1n ∑l=1mslkiTYilZi+∑i=1n ∑l=1mhlkiTDi2YilZi.

The objective of JR-LIP model is

(7)  min⁡ TC(Xij,Yil,Zi,ki,T) =∑i=1n ∑l=1mflYilZi+∑i=1n ∑l=1m ∑j=1mcljXijYilZi  +ST+∑i=1n ∑l=1mslkiTYilZi+∑i=1n ∑l=1mhlkiTDi2YilZis.t. ∑l=1mYil=1, ∀i,∑i=1nXij=1, ∀j,Xij−Zi≤0, ∀i,  ∀j,Yil−Zi≤0, ∀i,  ∀l.

The goal of this problem is to find the optimal *X*_*ij*_, *Y*_*il*_, *Z*_*i*_, *k*_*i*_, and *T* to minimize the TC.

## 3. Problem Solving Methodology

Since all involved costs in the model are associated with the location of the DC, the thinking of solving methodology is converting (7) to a function with *Y*_*il*_ firstly. Assuming that the index of opened DC (*Z*_*i*_), the customers assigned to the opened DC (*X*_*ij*_), and the replenishment policy of the opened DC (*k*_*i*_, *T*) are known, the problem we faced is where to locate these opened DCs (*Y*_*il*_) to minimize the total cost. For simplification, we denote that **A** is the set of opened DCs and **A**_*i*_ is the customer set of DCs *i*. Then the annual total cost can be rewritten as follows:

(8) TC(Yil)=∑i∈A ∑l=1mflYil+∑i∈A ∑l=1m ∑j∈AicljYil+ST +∑i∈A ∑l=1mslkiTYil+12∑i∈A ∑l=1mhlkiTDiYil.

For a given set {*X*, *Z*, *k*, *T*}, the total cost of each opened DC at each potential site (TC_il_*) can be calculated easily. Sorting the total cost of each opened DC of all potential sites by ascending, the optimal *Y*_il_* and TC_il_* of DC *i* is the first site in the list. If there are *p* DCs with the same best location index, the optimal *Y*_il_* and TC_il_* is obtained by comparing at least *p* combinations and at most *p*! combinations from the first site to the *p*th site in the list. Finally, sum TC_il_* and the optimal total cost TC*(*Y*_*il*_)  are obtained.

### 3.1. The Proposed Hybrid Self-Adapting DE Algorithm (HSDE)

Due to its easy implementation, quick convergence, and robustness, DE has turned to be one of the best evolutionary algorithms in a variety of fields [23–26]. However, the limitations on DE structure had inspired many scholars to improve upon DE by proposing modifications to the original DE fields [27].

#### 3.1.1. The works of Literature on DE's Modification

Neri and Tirronen [28] made a comprehensive study on DE's recent advances. They used twenty-four benchmark functions to survey the performance of eight DE-based algorithms. The comparative results showed that self-adapting parameters of DE (jDE) proposed by Brest et al. [29] were the effective and most simple improvement. Furthermore this modification also can avoid the manual parameter setting of *F* and CR.

The DE-based algorithms referred in Neri and Tirronen [28] were the modification for DE structure and many scholars also devoted to integrate other algorithm's superior scheme into DE. The most common integration is the combination of DE and GA operations. He et al. [30] combined GA and SQP operation into DE which is rather complex to be carried out for complex application cases. To avoid the limitation of one-to-one competition in DE, Lin [31] integrated the roulette wheel selection into DE. Further, Wang et al. [4] proposed a hybrid DE (HDE) which adopted a simpler selection scheme of GA named truncation selection. The numerical results showed that HDE is effective and can easily be applied in practical problems. The newest integration algorithm of DE that appeared recently is quantum-inspired differential evolution algorithms (QDEs) [32–35]. Since a quantum system with *n* qubits can represent 2^*n*^ states simultaneously, the population size of the algorithm can be smaller, even one individual [36]. However, due to the complex encoding and decoding scheme, it was not easy for complex practical problems.

Based on the above analysis, we propose a DE-based algorithm named HSDE by integrating the GA and self-adapting parameters of DE.

#### 3.1.2. The Operations of HSDE

The difference between HSDE and original DE is the structure of individual and selection operation.  Table 1 lists the notations used in the intelligent algorithm. The details are discussed as follows.

*(1) Individual Structure*. In HSDE, the control parameters mutation factor and crossover factor are related to the individual and evolution generation, not constant for all individuals in the whole evolution process. Based on the study of Brest et al. [29], the individual structure of HSDE is

(9) $$\begin{array}{c} x_{t}^{G}=\left\{x_{t,1}^{G},x_{t,2}^{G},\ldots ,x_{t,N_{d}}^{G},F_{t}^{G},\text{C}{\text{R}}_{t}^{G}\right\}, \end{array}$$

where *F*_*t*_^*G*^ and CR_*t*_^*G*^ are updated by the following formulation:

(10) $$\begin{array}{c} F_{t}^{G}=\{\begin{array}{cc} F_{\min}+{\text{rand}}_{1}\cdot F_{\max}, & \text{if}\;\;{\text{rand}}_{2}<\tau_{1},\\ F_{t}^{G-1}, & \text{otherwise}, \end{array} \end{array}$$

(11) $$\begin{array}{c} \text{C}{\text{R}}_{t}^{G}=\{\begin{array}{cc} {\text{rand}}_{3}, & {\text{if}\;\;\text{rand}}_{4}<\tau_{2},\\ \text{C}{\text{R}}_{t}^{G-1}, & \text{otherwise}, \end{array} \end{array}$$

where rand_1_,…, rand_4_ are randomly generated number with uniform distributed between 0 and 1; *τ*_1_ and *τ*_2_ are constant values which represent the probabilities of parameters' update; *F*_min⁡_ and *F*_max⁡_ are the minimum and maximum *F*, respectively.

*(2) Mutation*. The mutated vector is obtained according to the following equation:

(12) vt,1:NdG+1=xr1,1:NdG+FtG+1∗(xr2,1:NdG−xr3,1:NdG),r1≠r2≠r3≠t,

where *F*_*t*_^*G*+1^ is obtained by (10); *r*_1_, *r*_2_, *r*_3_, *t* ∈ [1,2,…, *N*_*p*_].

*(3) Crossover*. The crossover operation mixes the mutated vectors and the target vectors to increase the diversity of the parameter vector. The trial vector *u*_*t*_^*G*+1^ = {*u*_*t*1_^*G*+1^, *u*_*t*2_^*G*+1^,…, *u*_*tN*_*d*__^*G*+1^} can be generated as:

(13) $$\begin{array}{c} u_{tq}^{G+1}=\{\begin{array}{cc} v_{tq}^{G+1}, & \text{if}\;\;\text{rand}\left(q\right)\leq \text{C}{\text{R}}_{t}^{G+1}\;\;\text{or}\;\;q=\text{rand}\;n\left(t\right),\\ x_{tq}^{G}, & \text{otherwise}, \end{array} \end{array}$$

where *q* ∈ [1,2,…, *N*_*d*_]; rand(*q*)∈[0,1] is a randomly generated number with uniform distribution CR_*t*_^*G*+1^ is obtained from (11); rand *n*(*t*) ∈ [1,2,…, *N*_*d*_] is a randomly selected integer to ensure that the trial vector gets at least one gene from mutated vector [37].

(*4) Selection*. Considering that the one-to-one competing scheme may obsolete the superior individual exclusively, the advantage of GA in selection is utilized. The modified selection scheme is as follows: generate a new population {*x*_1_^*G*^, *x*_2_^*G*^,…, *x*_*N*_*p*__^*G*^, *u*_1_^*G*+1^, *u*_2_^*G*+1^,…, *u*_*N*_*p*__^*G*+1^} by combining the population {*x*_1_^*G*^, *x*_2_^*G*^,…, *x*_*N*_*p*__^*G*^} and {*u*_1_^*G*+1^, *u*_2_^*G*+1^,…, *u*_*N*_*p*__^*G*+1^}; compute the fitness value of the new population; sort the fitness value obtained; select half individuals those fitness value at the front as the next generation population {*x*_1_^*G*+1^, *x*_2_^*G*+1^,…, *x*_*N*_*p*__^*G*+1^}.

### 3.2. The Procedures of HSDE for the JR-LIP

**Figure 2:**
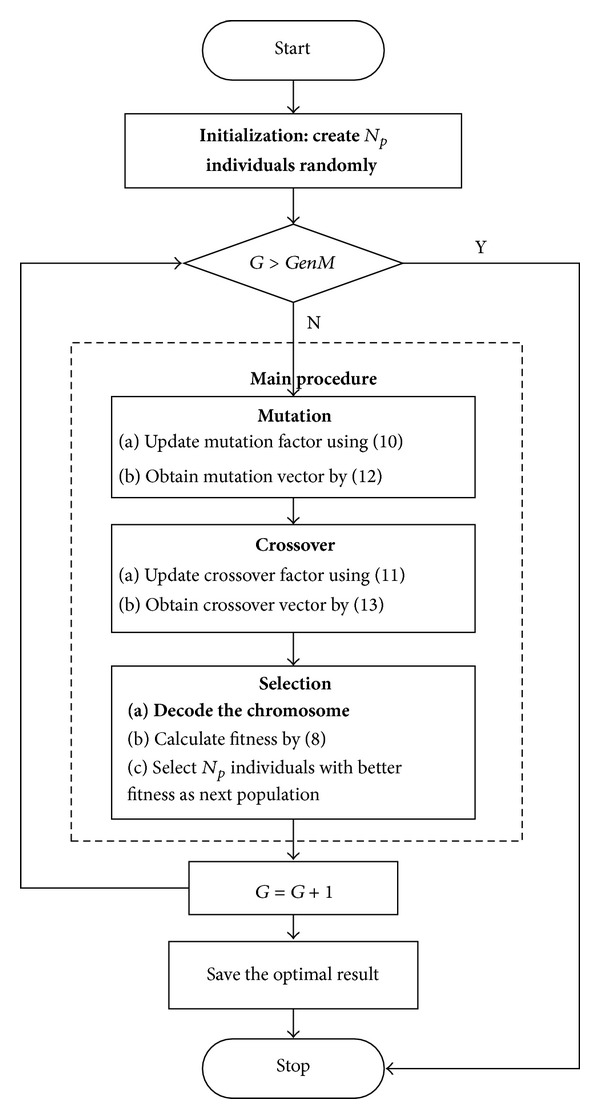
Flow chart of HSDE for the JR-LIP.

Figure 2 shows the flow chart of HSDE-based procedures for the JR-LIP. Since the common operations of HSDE have been described in Section 3.1, we only focus on the procedures for the specific problem in this section, that is, initialization and decoding scheme, which are bolded in Figure 2.

#### 3.2.1. Initialization

In the stage of initialization, we should determine the parameters of HSDE, the representation of chromosome, and the encoding and decoding scheme for the JR-LIP.

*The Parameters of HSDE*. Since the mutation factor and crossover factor for HSDE are encoded in the individual, the parameters that need to be determined are population size (*N*_*p*_), the number of iterations (*GenM*), the lower bound (*F*_min⁡_) and upper bound (*F*_max⁡_) of mutation factor, and the probabilities of parameters' update (*τ*_1_, and *τ*_2_). As suggested in Brest et al. [29], we set *F*_min⁡_ = 0.1, *F*_max⁡_ = 0.9, and *τ*_1_ = *τ*_2_ = 0.1. The values of *N*_*p*_ and *GenM* should be confirmed according to the practical problem as discussed in Section 4.

**Figure 3:**
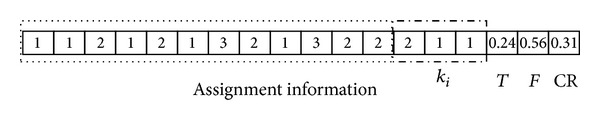
A decoded chromosome (*m* = 12, *n* = 3).

*The Representation of the Decoded Individual*. The proper representation of a solution plays a key role in the development of the HSDE. There are four parts included in the solution, the first part is the assignment information of customers; the second part is the replenishment frequency of DCs; the third part is the basic replenishment cycle time of DCs; the fourth part is the control parameters of HSDE. The dimension of the specific problem is *N*_*d*_ = *m* + *n* + 1 (*m* customers' assignment information; *n* DCs' ordering frequency; a basic ordering cycle time), and the total length of chromosome equals to *N*_*d*_ + 2.  Figure 3 shows a decoded chromosome of twelve customers, three DCs.

The first part of the decoded individual represents that customers 1, 2, 4, 6, and 9 are assigned to DC_1_; customers 3, 5, 8, 11, and 12 are assigned to DC_2_; customers 7, 10 are assigned to DC_3_. Thus, the decision variables *X*_*ij*_ and *Z*_*i*_ can be confirmed by assignment information. The rest of solution represents that DC_1_ replenishes its item at every 2*T* interval; DC_2_ and DC_3_ replenish their item at each *T* interval.

*The Initialization*. According to the representation of the decoded individual, the dimension of initial individual can be confirmed as *m* + *n* + 3. Then the values of *N*_*p*_ individuals with *m* + *n* + 3 dimension are generated between 0 and 1 randomly and then are mapped into practical values (see Figure 3) through decoding scheme.

#### 3.2.2. Decoding Scheme

The target of decoding is converting the initial chromosome to practical solution. Denote *x*_*t*,*q*_^*G*^ as the value of gene *q* of individual *t* in *G* generation; *p*_*t*,*q*_^*G*^ as the practical value by decoding *x*_*t*,*q*_^*G*^; *p*_*q*_^*L*^ as the lower bound of *x*_·,*q*_^*G*^; *p*_*q*_^*U*^ as the upper bound of *x*_·,*q*_^*G*^. Since the practical values of the first two parts of chromosome are integer, the formulation of decoding is as follows:

(14) pt,qG=round(pqL+xt,qG·(pqU−pqL)), q=1,…,Nd,t=1,…,Np;  G=1,…GenM.

The practical values of the last two parts of chromosome are between 0 and 1, since the value of gene can directly map into practical value.

From (14), we can see that the lower and upper bound is the key for decoding. It is obvious that the lower bound of opened DC and basic order cycle time are equal to 1. As to the upper bound, if the value is set too small, the optimal solution may be excluded; if the value is set too big, the search scope is enlarged which has directly impact on performance of algorithm. The maximum number of opened DCs (*n*) is usually decided according to the number and scope of customers; the upper bound of replenishment frequency (*k*_*i*_) is set to 15 which is almost 4 times of the maximum optimal replenishment frequency obtained by Silva and Gao [2].

## 4. Numerical Examples and Results Discussion

To verify the performance of the proposed HSDE, three numerical examples were designed. Another two intelligent algorithms, GAs and HDE which had been proven to be effective approaches for solving JRPs and JRDs [4, 19, 20], were chosen to compare with it. Example 1 is numerical optimization problems with benchmark functions; Example 2 is used to compare three algorithms by different sizes of the JR-LIPs; Example 3 is designed to observe the impact of different cost parameters on the optimal decision.

The decoding scheme of GA and HDE is the same with HSDE. Besides the number of populations (*N*_*p*_) and maximum number of iterations (*GenM*) and the rest of common parameters for three numerical examples are set as follows: (a) for HSDE, the parameters had been given in Section 3.2.1; (b) for HDE, crossover rate CR is set to 0.3, *F* is set to 0.6; (c) for GA, the probabilities of crossover and mutation are set to 0.9 and 0.1, respectively. The decisions for using these values are based on the experience from the works of literature [4, 29, 38].

### 4.1. Example 1: Comparative Study by Benchmark Functions

#### 4.1.1. Seven Benchmark Functions

In order to verify the performance of HSDE, seven well-known benchmark functions are used in the following experiments. To assure a relatively fair comparison, the functions were selected according to their different properties [29]. Functions *f*_1_–*f*_3_ are unimodal functions, *f*_4_ is a step function, and *f*_5_–*f*_7_ are multimodal functions which appear to be the most difficult class of problems for many intelligent algorithms. The details about seven test functions are listed in Table 2 (*L* denotes the dimension and *M* denotes the decision space of the problem).

#### 4.1.2. Comparative Results and Analysis

**Figure 4:**
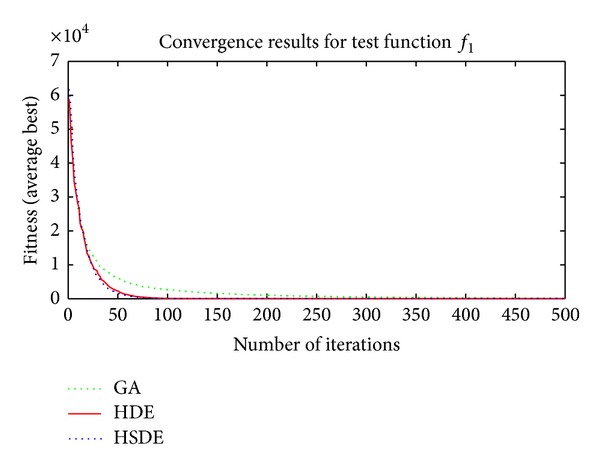
Convergence results of *f*_1_ (*L* = 30).

**Figure 5:**
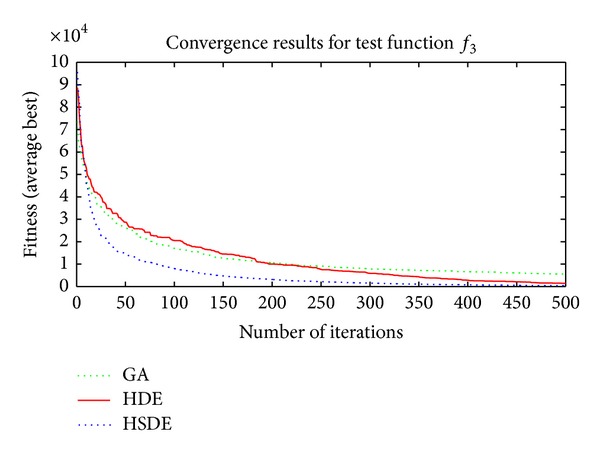
Convergence results of *f*_3_ (*L* = 30).

**Figure 6:**
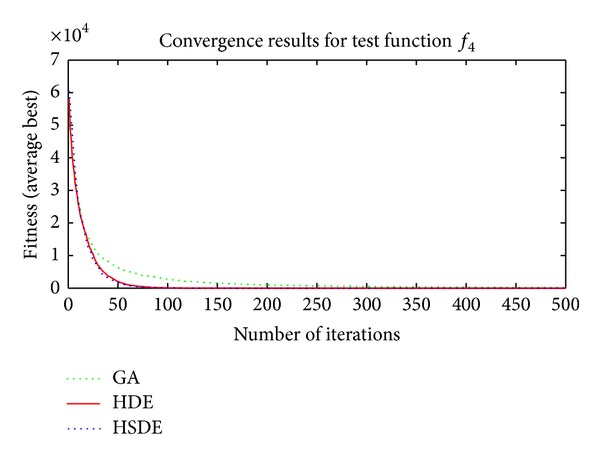
Convergence results of *f*_4_ (*L* = 30).

**Figure 7:**
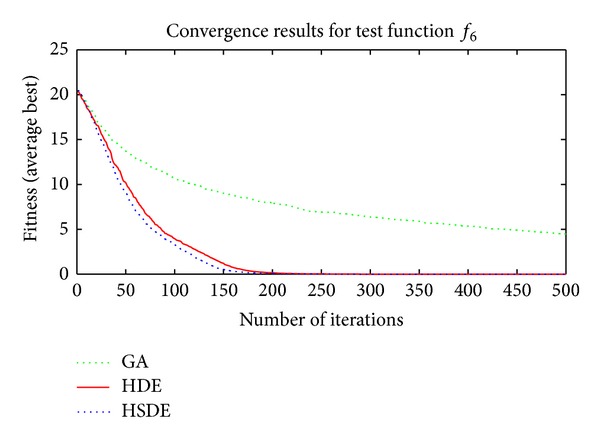
Convergence results of *f*_6_ (*L* = 30).

Three algorithms including HSDE, HDE, and GA are compared with two different dimensions, that is, the speed of convergence and the ability to obtain optimal solution. In all cases, the number of populations (*N*_*p*_) is 100; the maximum number of iterations (*GenM*) is 300 and 500 for *L* = 10 and *L* = 30, respectively. The average *f*_min⁡_ ± standard deviations of each algorithm for different test functions over 10 independent runs are shown in Tables 3 and 4. The best results are highlighted in bold face. Furthermore, in order to have an intuitive understanding about the convergence speed performance of each algorithm, Figures 4, 5, 6, and 7 present the average fitness curve of two unimodal functions (*f*_1_ and *f*_3_), step function *f*_4_ and multimodal function *f*_6_, with thirty dimensions.

Results in Tables 3 and 4 and Figures 4–7 show that

1. the ability to obtain optimal solutions of HSDE is better than HDE and GA, especially for functions with the large dimension (when *L* = 10, the cases to obtain best performance of HSDE, HDE, and GA are 6, 5, 0; for *L* = 30, the cases are 6, 2, 0, resp.);
2. although HSDE and HDE can both obtain the global optimum for *f*_4_ with thirty dimensions, the convergence curves presented in Figure 6 illustrate that HSDE has better convergence speed. In summary, the proposed HSDE has better performance for the large-scale optimization problems.

### 4.2. Example 2: Performance Comparison for the JR-LIPs

#### 4.2.1. Basic Data for the JR-LIP

In this section, three different scales of the JR-LIPs are used to test the performance of three intelligent algorithms. The part of basic data listed in Table 5 comes from Silva and Gao [2]. In all examples, the demand points were randomly located in a 50 × 50 square.

#### 4.2.2. Results and Analysis

**Figure 8:**
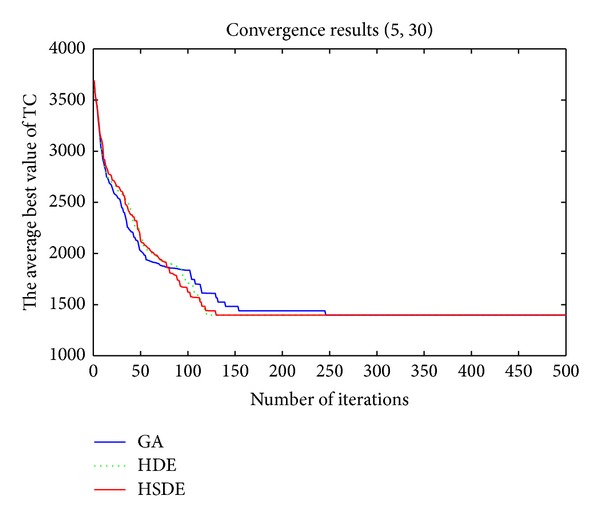
Convergence trend (*P*_5_30).

**Figure 9:**
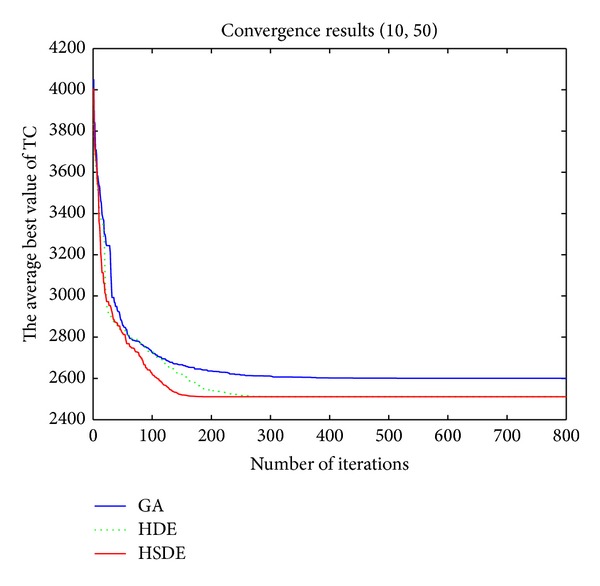
Convergence trend (*P*_10_50).

We denote *P*_*n*_*m* as the scale of problems. As for *N*_*p*_ of the HSDE and HDE, Storn and Price [38] proposed that the population size *N*_*p*_ ∈ [4*N*_*d*_, 10*N*_*d*_]; Nobakhti and Wang [39] and Wang et al. [40] suggested that *N*_*p*_ ∈ [2*N*_*d*_, 20*N*_*d*_] is rational. According to their experiments, we set *N*_*p*_ = 200, 300, and 450 for *P*_5_30, *P*_10_50, and *P*_20_100, respectively.  Table 6 reported the results of the average CPU times (Avg CPU times), the optimal total cost, the average minimum total cost for 20 times (Avg TC_min⁡_), and the ratio of finding the optimal total cost. Figures 8 and 9 showed the convergence trend of three algorithms (based on average fitness) for *P*_5_30 and *P*_10_50.

Results in Table 6 show that (1) both HSDE and HDE can find the optimal results with 100% for the smaller scale problem; (2) with the increasing of problem size, the performance of HSDE is better than HDE and GA whatever in CPU times and total cost. Furthermore, the running time of HSDE for *P*_10_50 (99 seconds) is superior to Silva and Gao [2] in which the running time is more than 270 seconds when *m* = 50.

Figures 8 and 9 also show that HSDE is more stable and suitable for the large-scale problem.

Section 4.2 mainly focuses on the performance of algorithm in handling different scales of JR-LIPs. The more detailed analysis about the impacts of cost parameters on the optimal decision is presented in Section 4.3.

### 4.3. Example 3: Impacts of Cost Parameters on Optimal Decision

The size of problem in this section is *P*_10_50 which is the middle scale of three problems in Section 4.2 and the optimal TC can be found at each running by HSDE and HDE. GA is not used in this section due to its inferior performance (the ratio to find optimal TC is 0%). Tables 7 and 8 show the computational results by varying the values of inventory holding cost and major ordering cost. From the tables we can see clearly the impacts of cost parameters on DC selection, replenishment frequency (*k*_*i*_), location costs (*C*_*L*_), replenishment costs (*C*_*R*_), and total running time of algorithms.

The comparative results of Tables 7 and 8 show the following useful conclusions.

1. The robustness of HSDE is better than HDE.
2. When inventory holding cost and major ordering cost vary from −40% to 20%, the optimal number of DCs is always three. Moreover, the location sites of DCs have little change (Δ*S* = 20%), and the varied direction of replenishment cost is the same with the cost parameters.
3. DCs have more motivation to order item jointly when the major ordering cost S increases (*k*_1_ = *k*_2_ = *k*_3_ = 1 indicates that DCs replenish jointly in each order). This observation is consistent with the principle of JR policy.

## 5. Conclusions and Future Research

In this paper, we proposed an effective intelligent algorithm for a modified joint replenishment and location-inventory (JR-LIP) model. The objective of the JR-LIP is to determine the number and locations of DCs, the assignment decision, and replenishment policy to minimize the total system cost. To handle this NP-hard problem effectively, an intelligent algorithm named HSDE is designed to solve the proposed model. To verify the effectiveness of HSDE, GA and HDE were chosen to be compared with it by benchmark functions tests and numerical examples. We can easily come to useful conclusions and managerial insight as follows.

1. Results of benchmark functions tests show the good ability of HSDE in handling the large-scale problems. When the dimension of test function is 10, HSDE and HDE have the same precision, while the dimension is 30, HSDE has the higher precision and faster speed than HDE.
2. The similar conclusion can be obtained from example 2. The rate of convergence obtained by HSDE and HDE is both 100% when *m* = 30 and *m* = 50, whereas when *m* = 100, the rate of HDE is 0% and HSDE is 45%.
3. Example 3 illustrates the impacts of cost parameters on the optimal decision and reveals that when the major ordering cost is bigger, DCs have more incentive to replenish jointly for sharing related costs.

All numerical examples verify that the HSDE is an easy and effective algorithm to handle the JR-LIP. To our best knowledge, this is the first time to use the improved DE-based algorithm to solve this NP-hard problem.

In our model, the demand is constant which generated from a uniform distribution, and there is no capacity limitation. These assumptions are not required. The future direction about this problem includes relaxing the assumption to match real-world scenario and looking for more quickly and efficiently solving method.

## Figures and Tables

**Table 1:** HSDE's notations.

Notation	Explanation
*N*_*p*_	Population: the number of individuals
*N*_*d*_	The dimension of the specific problem
*GenM *	The maximum generation for evolution
*x*_*t*_^*G*^	The target vector of individuals *t *in* G* generation
*v*_*t*_^*G*^	The mutated vector of individuals *t* in *G* generation
*u*_*t*_^*G*^	The trial vector of individuals *t* in *G* generation
*F*_*t*_^*G*^	Mutation factor of individuals *t* in *G* generation
CR_*t*_^*G*^	Crossover factor of individuals *t* in *G* generation

**Table 2:** Benchmark functions.

Test functions	*L *	*M *	Global optimum (*f*_min_)
*f*_1_(*x*) = ∑_*l*=1_^*L*^ *x*_*l*_^2^	10, 30	[−100,100]^*L*^	0
*f*_2_(*x*) = ∑_*l*=1_^*L*^|*x*_*l*_| + ∏_*l*=1_^*L*^|*x*_*l*_|	10, 30	[−10,10]^*L*^	0
*f*_3_(*x*) = ∑_*l*=1_^*L*^(∑_*g*=1_^*l*^ *x*_*g*_)^2^	10, 30	[−100,100]^*L*^	0
*f*_4_(*x*) = ∑_*l*=1_^*L*^(⌊*x*_*l*_ + 0.5⌋)^2^	10, 30	[−100,100]^*L*^	0
$f_{5}(x)=\sum_{l=1}^{L}-x_{l}\sin\left(\sqrt{\left|x_{l}\right|}\right)$	10, 30	[−500,500]^*L*^	−12569.5
$\begin{array}{c} f_{6}(x)=-20\exp\left(-0.2\sqrt{\left(\sum_{l=1}^{L}x_{l}^{2}\right)/L}\right)\\ -\exp\left(\left(1/L\right)\sum_{l=1}^{L}\cos2\pi x_{l}\right)+20+e \end{array}$	10, 30	[−32,32]^*L*^	0
$f_{7}(x)=\left(1/4000\right)\sum_{l=1}^{L}x_{l}^{2}-\prod_{l=1}^{L}\cos\left(x_{l}/\sqrt{l}\right)+1$	10, 30	[−600,600]^*L*^	0

**Table 3:** Results of seven functions with three algorithms (*L* = 10).

Function	HSDE	HDE	GA
*f*_ 1_	0	0	0.0714 ± 0.0341
*f*_ 2_	0	0	0.0482 ± 0.0090
*f*_ 3_	1.4868**e** − 06 ± 5.3600*e* − 06	8.3092*e* − 06 ± 2.6265*e* − 05	81.1709 ± 57.6638
*f*_ 4_	0	0	0.6928 ± 0.2275
*f*_ 5_	−4.1646*e* + 03 ± 53.1791	−4.1898**e** + 03 ± 9.5869*e* − 13	−3.7682*e* + 03 ± 0.3011
*f*_ 6_	8.8820**e** − 16 ± 2.0788**e** − 31	8.8820**e** − 16 ± 2.0788**e** − 31	0.1348 ± 0.0492
*f*_ 7_	0.0138 ± 0.0107	0.0143 ± 0.0084	0.1653 ± 0.0395

**Table 4:** Results of seven functions with three algorithms (*L* = 30).

Function	HSDE	HDE	GA
*f*_ 1_	1.4139**e** − 13 ± 1.2000*e* − 13	3.8990*e* − 12 ± 1.5278*e* − 12	133.5903 ± 37.2994
*f*_ 2_	8.3180**e** − 09 ± 4.2406*e* − 09	5.6406*e* − 08 ± 2.3898*e* − 08	2.7869 ± 0.4786
*f*_ 3_	5.1928**e** + 02 ± 1.1432*e* + 03	1.4403*e* + 03 ± 890.9681	3.8840*e* + 03 ± 1.7548*e* + 03
*f*_ 4_	**0**	**0**	148.8000 ± 43.3585
*f*_ 5_	−1.1944*e* + 04 ± 439.6241	−1.2475**e** + 04 ± 93.0797	−1.2417*e* + 04 ± 35.5129
*f*_ 6_	7.5180**e** − 08 ± 3.3095*e* − 08	3.9619*e* − 07 ± 1.1968*e* − 07	4.1132 ± 0.3369
*f*_ 7_	0.0012 ± 0.0032	0.0016 ± 0.0042	2.1764 ± 0.3635

**Table 5:** Parameters for test problems.

Parameters		Value
Common parameters		
*D*_*j*_, *j* = 1, 2, …, *m*	Annual demand rate	*U* (80, 800)
*S*	Major ordering cost	45
*s*_*l*_, *l* = 1, 2, …, *m*	Minor ordering cost	*U* (1, 10)
*h*_*l*_, *l* = 1, 2, …, *m*	Annual inventory holding cost	*U* (0, 1)
*f*_*l*_, *l* = 1, 2, …, *m*	Fixed location cost	*U* (400, 800)
Changed parameters		
*n*	Maximum number of opened DCs	5, 10, 20
*m*	Number of customers (potential sites of DCs)	30, 50, 100

**Table 6:** Comparison of the results for different scales.

*P*_*n*_*m*	Algorithm	Avg. CPU times	Optimal TC	Avg. TC_min⁡_	Ratio of finding the optimal TC
*P*_5_30	HSDE	17.6	1397.4	1397.4	100%
	HDE	17.3	1397.4	1397.4	100%
	GA	19.2	1397.4	1397.4	100%

*P*_10_50	HSDE	99.0	2511.2	2511.2	100%
	HDE	105.1	2511.2	2511.2	100%
	GA	114.1	3064.8	3126.0	0%

*P*_20_100	HSDE	613.2	4444.7	4709.2	45%
	HDE	689.9	7318.3	7846.4	0%
	GA	742.3	8539.4	9497.5	0%

**Table 7:** Comparative results using three algorithms under different *h*_*l*_.

Δ*h*_*l*_ (%)	Algorithms	Location sites (DCs)	CPU time	*k*_*i*_	*C*_*L*_	*C*_*R*_	*C*_*L*_ + *C*_*R*_
−40	HSDE	36, 39, 32	85.6	1,1, 1	2174.7	298.7	2473.4
	HDE	32, 39, 36	81.0	1,1, 1	2174.7	298.7	2473.4
−20	HSDE	32, 39, 36	89.5	1,1, 1	2177.0	342.3	2519.3
	HDE	32, 39, 36	88.3	1,1, 1	2177.0	342.3	2519.3
20	HSDE	39, 36, 32	106.4	1,1, 1	2177.0	419.2	2596.2
	HDE	32, 39, 36	113.6	1,1, 1	2177.0	419.2	2596.2

**Table 8:** Comparative results using three algorithms under different *S. *

Δ*S* (%)	Algorithms	Location sites (DCs)	CPU time	*k*_*i*_	*C*_*L*_	*C*_*R*_	*C*_*L*_ + *C*_*R*_
−40	HSDE	31, 9, 3	105.4	2, 1, 1	2888.9	394.5	3283.4
	HDE	3, 31, 9	109.9	2, 2, 1	2881.4	403.0	3284.4
−20	HSDE	49, 31, 3	96.7	1, 1, 1	2951.2	358.2	3309.4
	HDE	3, 31, 9	106.8	1, 1, 1	2924.2	401.2	3325.4
20	HSDE	31, 49, 3	107.9	1, 1, 1	2966.1	429.2	3395.3
	HDE	31, 49, 3	111.5	1, 1, 1	2966.1	429.2	3395.3
